# Interplay Between Non-Coding RNAs and Programmed Cell Death Proteins

**DOI:** 10.3389/fonc.2022.808475

**Published:** 2022-03-23

**Authors:** Soudeh Ghafouri-Fard, Bashdar Mahmud Hussen, Mahdi Mohaqiq, Hamed Shoorei, Aria Baniahmad, Mohammad Taheri, Elena Jamali

**Affiliations:** ^1^ Department of Medical Genetics, School of Medicine, Shahid Beheshti Universality of Medical Sciences, Tehran, Iran; ^2^ Department of Pharmacognosy, College of Pharmacy, Hawler Medical University, Erbil, Iraq; ^3^ School of Advancement, Centennial College, Toronto, ON, Canada; ^4^ The Ottawa Hospital Research Institute, University of Ottawa, Ottawa, ON, Canada; ^5^ Department of Anatomical Sciences, Faculty of Medicine, Birjand University of Medical Sciences, Birjand, Iran; ^6^ Institute of Human Genetics, Jena University Hospital, Jena, Germany; ^7^ Urology and Nephrology Research Center, Shahid Beheshti Universality of Medical Sciences, Tehran, Iran; ^8^ Skull Base Research Center, Loghman Hakim Hospital, Shahid Beheshti Universality of Medical Sciences, Tehran, Iran

**Keywords:** lncRNA, miRNA, programmed cell death protein, expression, biomarker

## Abstract

Programmed cell death (PDCD) family of proteins includes at least 12 members, function of seven of them being more investigated. These members are PDCD1, PDCD2, PDCD4, PDCD5, PDCD6, PDCD7 and PDCD10. Consistent with the important roles of these proteins in the regulation of apoptosis, dysregulation of PDCDs is associated with diverse disorders ranging from intervertebral disc degeneration, amyotrophic lateral sclerosis, immune thrombocytopenia, type 1 diabetes, congenital hypothyroidism, Alzheimer’s disease to different types of cancers. More recently, the interaction between non-coding RNAs and different members of PDCD family is being discovered. In the current study, we described the functional interactions between PDCDs and two classes of non-coding RNAs, namely microRNAs (miRNAs) and long non-coding RNAs (lncRNAs). miR-21 and miR-183 are two miRNAs whose interactions with PDCDs have been assessed in different contexts. The lncRNAs interaction with PDCDs is mainly assessed in the context of neoplasia indicating the role of MALAT1, MEG3, SNHG14 and LINC00473 in this process.

## 1 Introduction

Programmed cell death (PDCD) has been observed as an important phenomenon during insect development about seven decades ago (1). Afterwards, several equivalent cell death phenomena have been recognized in vertebrates including human and this process has been retitled as apoptosis (2). This cellular process in contributes in eradication of foreign bodies and abnormal cells. Moreover, it has essential roles in the development of organisms, the homeostasis of the internal milieu and organ development. Abnormal regulation of apoptosis is strictly associated with immune disorders, developmental abnormalities, and cancers (3). Apoptosis is regulated by two types of proteins with one of them inhibiting cell death and the other group initiating this process. Genes regulating apoptosis are highly conserved between various species and genera (4). Different studies have shown wide expression of PDCD gene family members in normal adult tissues (4).

**Figure 1:**
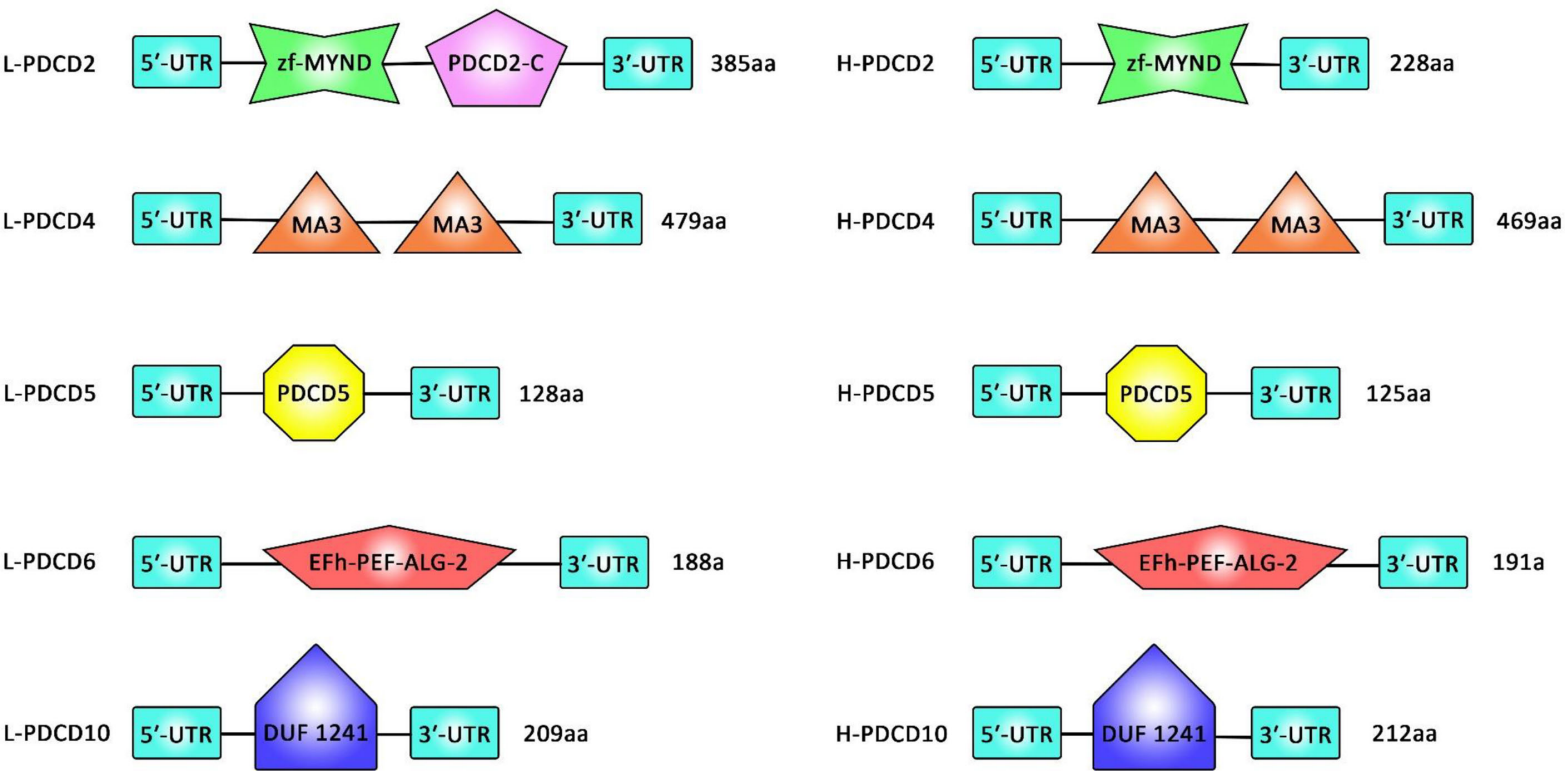
A comparison between the sequence of the domains of PDCD family genes (PDCD2, PDCD4, PDCD5, PDCD6, and PDCD10) in Lamprey and human.

A comprehensive analysis of several PDCD gene family members in vertebrates and assessment of their sequences as well as alignment steps and 3D structure analyses has revealed no similarity in the structural domains between the PDCD family genes. In fact, PDCD4 family genes have no structural similarity but contain some conserved amino acid sequences (or so call motifs). Lamprey PDCD genes have been found to be highly homologous with the corresponding human PDCD genes. **Figure 1** shows the conserved motifs between lamprey and human PDCD genes.

Assessment of motifs in the PDCD proteins has resulted in identification of 16 distinct motifs in these proteins. All lamprey PDCD proteins have been shown to contain motif 13. PDCD2 proteins have been found to contain motifs 7, 9, 13, 15, and 16. Besides, motifs 4, 8, 10, 11, 12, 13, and 14 have been present in PDCD4 proteins. Finally, motifs 1, 2, and 13 have been detected in PDCD5 proteins. This bioinformatics analysis has indicated that individual motifs might contribute in the biological activity of PDCD proteins. Phylogenetic studies have also verified the conserved evolution of each PDCD gene in vertebrates (4).

PDCD1 has an important role in the regulation of the immune system responses and induction of self-tolerance through inhibition of activity of T cells (5). PDCD2 participates in the development of embryo and differentiation of stem cells (6, 7). PDCD4 participates in tumor evolution, cancer progression and metastatic processes (8, 9). In fact, PDCD4 participates in the regulation of transcription, translation, apoptototic pathways, and regulation of various signal transduction pathways (10). Expression of PDCD5 has been found to be increased in TF-1 cells undertaking apoptosis (11). PDCD6 has been shown to be involved in cell proliferation and death (12). PDCD7 participates in apoptosis induced by glucocorticoids and staphylococci (13). PDCD10 has diverse roles in proteins synthesis, apoptotic pathways, cell proliferation, and induction of tumors (14, 15).

Moreover, expression levels of PDCD genes have been demonstrated to be altered in tumor samples and cancer cell lines (16, 17). At present, there is no evidence demonstrating a conclusive link between members of PDCD gene family (4). Yet, assessment of transcriptomic data has shown remarkable alterations in expression of the PDCD gene family following treatment with certain drugs (18). Although 12 members of this family have been identified in the human genome, seven main genes, namely PDCD1 (alternatively named as PD-1), PDCD2, PDCD4, PDCD5, PDCD6, PDCD7 and PDCD10 are in the focus of mechanistical investigations (4). PD-1 has two ligands, namely PD-L1 and PD-L2 which are present on the surface of dendritic cells or macrophages (19).

More recently, the interaction between non-coding RNAs and different members of PDCD family is being discovered. In the current study, we described the functional interactions between PDCDs and two classes of non-coding RNAs, namely microRNAs (miRNAs) and long non-coding RNAs (lncRNAs). miRNAs are a group of small-sized non-coding RNAs that regulate gene expression at post-transcriptional level through base-pairing with mRNAs and inducing mRNA degradation or suppressing mRNA translation. LncRNAs can regulate gene expression at different levels through epigenetic mechanisms, modification of RNA stability and interaction with several types of biomolecules.

## 2 miRNAs and PDCD

miRNAs are transcripts with sizes about 22 nucleotides that regulate gene expression through binding with different regions of mRNAs, particularly their 3’ UTR (20). In addition to transcript degradation and translational suppression, miRNAs have been found to induce translation or modulate transcription (20). Most studies have shown that miRNAs regulate PDCD expression through binding with 3’ UTR of PDCD transcripts. This kind of interaction has been predicted by bioinformatics approaches and verified through luciferase assay. Binding of miRNAs with this region of PDCD transcript leads to down-regulation of expression of PDCDs. The degree of miRNA response elements complementarity defines whether the target mRNA is subjected to AGO2-dependent slicing or its translation is inhibited by miRNA-induced silencing complex and target mRNA decay (20). It has also been shown that in some situations, miRNAs may activate translation of target mRNAs or regulate transcription (20).

### 2.1 Interaction Between miRNAs and PDCD in Non-Neoplastic Disorders

#### 2.1.1 Heart Diseases

Suppression of PDCD4 by miR-21 has been shown to contribute to the induction of fibroblastoid features during cardiac injury leading to cardiac fibrosis, since contributing in the pathogenesis of fibrogenic cardiac injury. In fact, pro-fibrogenic incitements, particularly TGF-β, can promote progression of epithelial-mesenchymal transition (EMT) in epicardial mesothelial cells, resulting in alterations in miRNAs signature, specifically expression of the pleiotropic miR-21. Ectopic expression of this miRNA has noticeably stimulated the fibroblast-like features causing by fibrogenic EMT, while miR-21 antagonism has suppressed this effect (21). Another study has shown up-regulation of miR-21 in macrophages in response to elevation of glucose levels. Elevation of glucose levels has been shown to stimulate apoptosis of macrophages. Concurrent inhibition of miR-21 and elevation of glucose concentrations results in enhancement of cell apoptosis (citation). Taken together, miR-21 levels have been changed by high glucose concentrations in macrophages, exerting a protective impact against glucose-induced macrophage apoptosis through suppression of PDCD4 expression (22). miR-208a-3p is another miRNA that could suppress expression of PDCD4, thus affecting autophagy in rat cardiomyoblasts (23). Meanwhile, the inhibitory effects of miR-499-5p on PDCD4 have been shown to decrease cardiomyocytes apoptosis and reduce myocardial infarct size (24). Finally, miR-613 through targeting PDCD10 could suppresses ischemia/reperfusion-induced cardiomyocyte apoptosis *via* regulating the PI3K/AKT signaling pathway (25). **Table 1** shows the interaction between miRNAs and PDCD in heart diseases.

**Table 1:** Interaction between miRNAs and PDCD in heart diseases.

Disease	miRNA	Animal/human	Cell lines	Targets	Pathways	Function	Ref
Cardiac Fibrosis	miR-21	Adult SD rats, female C57/BL6 mice	–	PDCD4, SPRY1, IL-1β, TNF-α, TGF-β, α-SMA, Slug, E-cadherin	–	Upregulation of miR-21 by targeting PDCD4 and SPRY1 could increase fibrogenic EMT of epicardial mesothelial cells.	(21)
Cardiovascular Disease	miR-21	–	Raw 264.7	PDCD4, Caspase-3	–	Upregulation of miR-21 which is induced by high levels of glucose could reduce macrophage apoptosis by targeting PDCD4.	(22)
Cardiac Hypertrophy	miR-208a-3p	–	H9c2, 293T	PDCD4, ATG5, LC3BII/I, P62		miR-208a-3p could increase the autophagic activity *via* PDCD4-ATG5 pathway in Ang II-induced H9c2 cardiomyoblasts in rats.	(23)
Myocardial I/R Injury	miR-613	–	H9c2	PDCD10, PDCD10, MDA, CHOP, GRP78, Bcl-2, Caspase-3/12, Bax, Cytochrome-c	p-Akt, p-JNK	Upregulation of miR-613 by targeting PDCD10 could suppress I/R-induced cardiomyocyte apoptosis *via* regulating the PI3K/AKT signaling pathway.	(25)
Acute Myocardial Infarction (AMI)	miR-499-5p	Male SD rats	–	PDCD4	–	Upregulation of miR-499-5p could suppress cardiomyocytes apoptosis and myocardial infarct size of AMI *in vitro* and *in vivo*.	(24)

#### 2.1.2 Polycystic Ovary Syndrome (PCOS)

Polycystic ovary syndrome (PCOS) is the utmost frequent female endocrine disease (26) being characterized by the presence of ovarian cysts, chronic anovulation, and clinical or biological signs of hyperandrogenism. People with PCOS may experience irregular menstrual periods, heavy periods, excess hair, acne, pelvic pain, difficulty getting pregnant, and patches of thick, darker, velvety skin (3) The primary characteristics of this syndrome include: hyperandrogenism, anovulation, insulin resistance, and neuroendocrine disruption Expression of miR-16 expression has been shown to be decreased in ovarian cortex tissues and serums of PCOS patients, parallel with up-regulation of PDCD4. Mechanistically, miR-16 enhances cell proliferation, facilitates cell cycle progression, and suppresses apoptosis in granulosa cells through inhibiting expression of PDCD4. Forced over-expression of PDCD4 has stopped the impact of miR-16 on growth and apoptosis of granulosa cells. Moreover, testosterone could reduce expression level of miR-16 and enhance PDCD4 levels, therefore hindering cell growth and enhancing apoptosis of granulosa cells (27). Another study in steroidogenic human ovarian granulose-like tumor cell line has shown that upregulation of miR-155 by targeting PDCD4 and regulating PI3K/AKT and JNK pathways could enhance proliferation, migration, and invasion of cells. Since miR-155 has been found to be up-regulated in PCOS samples, miR-155/PCDC4 axis might be involved in the pathogenesis of PCOS (28). **Table 2** shows the interaction between miRNAs and PDCD in PCOS.

**Table 2:** Interaction between miRNAs and PDCD in polycystic ovary syndrome (PCOS).

miRNA	Animal-human	Cell lines	Targets	Pathways	Function	Ref
miR-16	Female Wistar rats/Human: 19 pairs of PCOs and normal healthy women	GCs	PDCD4, PCNA, caspase-3	–	Upregulation of miR-16 by targeting PDCD4 could enhance ovarian GCs proliferation and suppress apoptosis in PCOS.	(27)
miR-155	20 pairs of PCOS tissues and normal tissues	KGN	PDCD4, c-Myc, p21 Cyclin-D1, Vimentin, Caspase-3/9, p53, MMP-2/9	PI3K/AKT, JNK	Upregulation of miR-155 by targeting PDCD4 and regulating PI3K/AKT and JNK pathways could enhance proliferation, migration, and invasion in KGN cells.	(28)

#### 2.1.3 Other Non-Neoplastic Disorders

**Figure 2:**
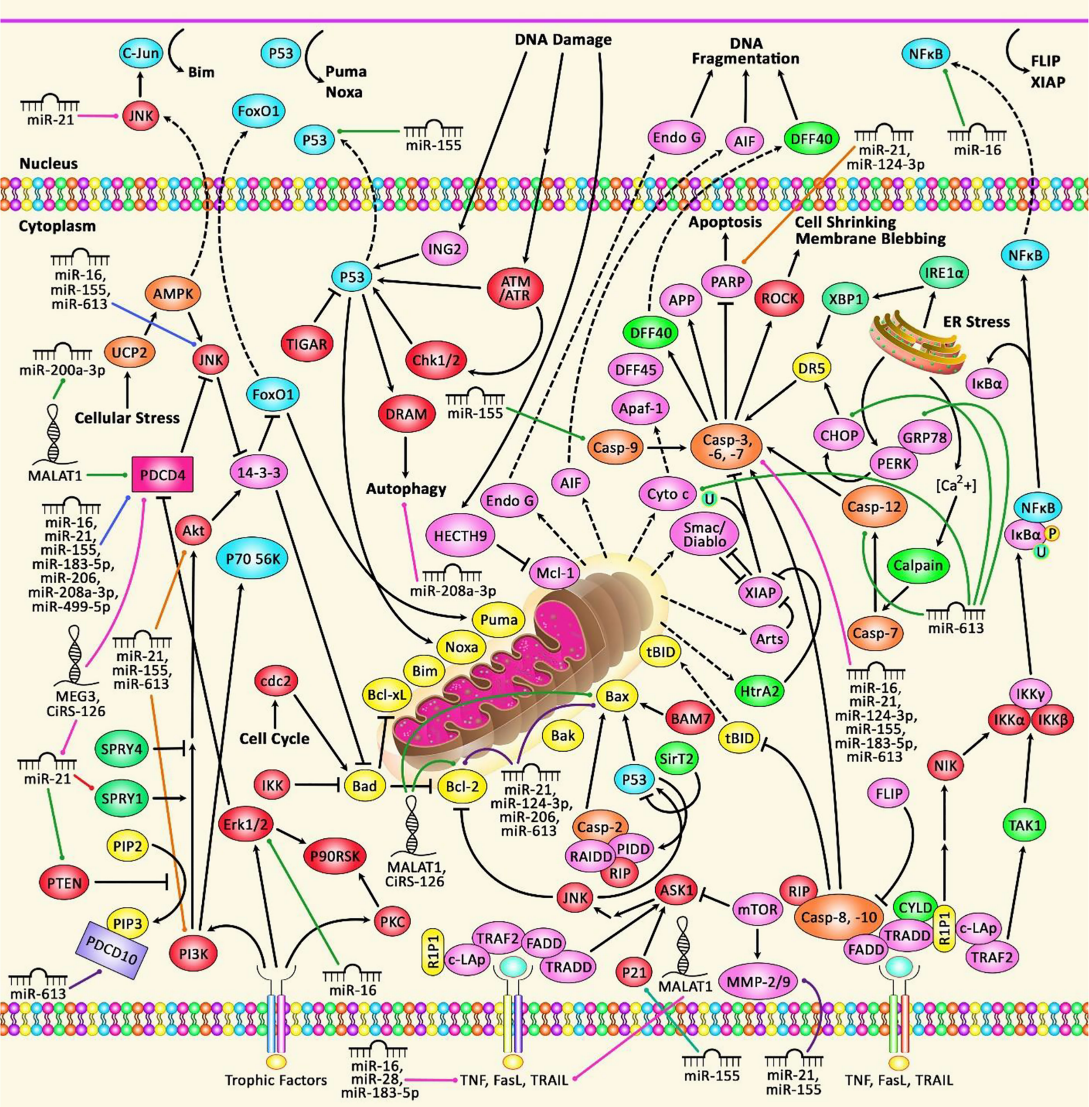
A schematic diagram of the functional interactions between PDCDs proteins and various ncRNAs in non-neoplastic disorders. Apoptosis, a gene-controlled process of programmed cell death (PCD), is a cascade which is induced in normal cells under physiological conditions and pathological stress. Dysregulation of apoptosis can result in uncontrolled cell proliferation that can play a significant role in causing various non-neoplastic disorders including heart diseases, polycystic ovary syndrome, and several immune disorders. The death receptors include Fas receptors, TNF receptors, and TRAIL receptors. As a surface receptor, TNF-R1 can interact with TNF to induce the recruitment of adaptor proteins FADD and TRADD, which can recruit a series of downstream factors, such as Caspase-8, that is a key modulator of the extrinsic cascade, eventually leading to cell apoptosis. In the intrinsic cascade, the functional outcome of the pro-apoptotic pathway is mitochondrial membrane perturbation and release of cytochrome c in the cytoplasm, where it can create a complex with APAF1 and the inactive form of caspase-9. In order to trigger the activation of caspase-9, this complex hydrolyzes adenosine triphosphate. Consequently, the initiator caspase-9 can cleave and upregulate the executioner caspases-3/6/7, leading to cell apoptosis (42, 43). Growing evidence suggests that dysregulation of PDCDs is associated with several non-neoplastic disorders and importantly their interaction with different ncRNAs (miRNAs and lncRNAs) is also detected.

The impact of miRNAs on expression of PDCD is also involved in a variety of other non-neoplastic disorders. For instance, in the context of intervertebral disc degeneration, up-regulation of miR-21 could promote proliferation of human degenerated nucleus pulposus cells through regulating PDCD4 expression, enhancing phosphorylation of c-Jun protein, and activating AP-1-dependent transcription of MMP-2/9 (29). miR-183-5p, a highly expressed miRNA in neurons has been shown to inhibit expression of PDCD4. Neuronal expression of this miRNA is instantly increased in response to treatment with hydrogen peroxide, tunicamycin or TNF-α. Its up-regulation enhances survival of neurons under stress situations, while its silencing leads to death of neurons. In fact, miR-183-5p synchronizes apoptosis and necroptosis mechanisms through its direct interactions with PDCD4 and RIPK3. This function shields neurons against cell death under stress situations. Consistently, expression of miR-183-5p has been found to be reduced in amyotrophic lateral sclerosis patients and animal models enhances supporting the role of this miRNA in regulation of motor neuron survival (30). Another set of *in vivo* and *in vitro* experiments has shown that down-regulation of miR-21 could increase fibroblast apoptosis and prevent knee scar adhesion though influencing PDCD4 expression. In fact, miR-21 attenuates the impact of mitomycin on decreasing the number of fibroblasts through down-regulating PDCD4 levels (31). miR-16 is another miRNA that targets PDCD4. Through this route, miR-16 can inhibit activation of inflammatory macrophages during the atherosclerotic process through the MAPK and NF-κB pathways (32). In the context of steroid-induced avascular necrosis of femor, miR−206 by targeting PDCD4 could reduce cell viability and proliferation, and enhance apoptosis (33).

hsa-miR-424-5p has been found to bind with PD-1 stimulating immune responses through the mTORC signal transduction, thus participating in the pathoetiology of type I diabetes (34). Similarly, up-regulation of miR-28 by targeting PD-1 and regulating cytokine secretion could modulate exhaustive differentiation of T cells (35). **Table 3** shows interaction between miRNAs and PDCD in diverse non-neoplastic disorders. **Figure 2** demonstrates that aberrant expression of PDCD-interacting ncRNAs could play an effective role in causing several non-neoplastic disorders.

**Table 3:** Interaction between miRNAs and PDCD in other non-neoplastic disorders.

Disorder	miRNA	Animal-human	Cell lines	Targets	Pathways	Function	Ref
–	miR-21-5p	–	L02	PDCD4, ROS, MMP, MRCC I/II	–	Upregulation of miR-21-5p by ROS through targeting PDCD4 could regulate the proliferation and apoptosis in L02 hepatocytes.	(36)
–	miR-28	C57BL/6 mice	B16F10	PD1, Foxp3^+^, BTLA, TIM3, IL-2, TNF-α	–	Upregulation of miR-28 by targeting PD-1 and regulating cytokine secretion could modulate exhaustive differentiation of T cells.	(35)
Intervertebral Disc Degeneration (IDD)	miR-21	20 IDD patients and 5 healthy control	–	PDCD4, MMP-2, MMP-9	c-Jun	Upregulation of miR-21 by regulating PDCD4 expression, enhancing phosphorylation of c-Jun protein, and activating AP-1-dependent transcription of MMP-2/9 could promote the proliferation of human degenerated NP cells.	(29)
Amyotrophic Lateral Sclerosis (ALS)	miR-183-5p	–	Neuro2a, NSC-34	PDCD4, TNF-α, RIPK3, Caspase-3	–	Upregulation of miR-183-5p by targeting PDCD4 could enhance the survival rate of neurons under stress conditions in ALS cell lines.	(30)
Knee Scar Adhesion	miR-21	Rabbit	293T	PDCD4, PARP, Bax, Bcl-2	–	Downregulation of miR-21 could increase fibroblast apoptosis and prevent knee scar adhesion through influencing expression of PDCD4 *in vivo* and *in vitro*.	(31)
Steroid−Induced Avascular Necrosis Of Femoral Head (SANFH)	miR-206	15 SANFH and 15 healthy control specimens	hFOB1.19, 293T	PDCD4, ALP, Bax, Bcl-2	–	Upregulation of miR−206 by targeting PDCD4 could reduce cell viability and proliferation, and enhance apoptosis in hFOB1.19 cells.	(33)
Immune Thrombocytopenia (ITP)	miR-155-5p	Female CBA mice/Human: 42 patients with ITP and 30 healthy volunteers	PBMCs, 293T	PD1, PDL1, SOCS1, IL-4, IL-10, IL-17A, TGF-β1	–	Downregulation of miR-155-5p could induce the PD1/PDL1 pathway-mediated macrophage M2 polarization and suppress ITP progression *via* targeting SOCS1.	(37)
Contrast-Induced Acute Kidney Injury (CI-AKI)	miR-21	–	HK-2, 293T	PDCD4, Bcl-2, Bax	–	Upregulation of miR-21 by targeting PDCD4 could suppress HK-2 cell apoptosis.	(38)
Type 1 Diabetes (T1D)	miR-424-5	Male SD rats	–	PD-1, T-bet, CXCR3, STING, IGF-1, SHP2, Rheb, Rictor	mTORC	Upregulation of miR-424-5p by targeting PD-1 signaling molecules could result in the immune response in T1D.	(34)
Congenital Hypothyroidism (CH)	miR-124-3p	Pregnant SD rats	–	PDCD6, PARP, Caspase-3, Bcl−2, Bax	–	Upregulation of miR-124-3p by targeting PDCD6 could suppress the progression of CH.	(39)
Alzheimer’s Disease (AD)	miR-21	–	SH-SY5Y	PDCD4, amyloid-β, GSK-3β, Bcl-2, Bax	PI3K/AKT	Upregulation of miR-21 by targeting the PDCD4/PI3K/AKT/GSK-3β pathway could reduce apoptosis in SH-SY5Y cells.	(40)
Atherosclerosis	miR-16	ApoE^-/-^ mice with a C57BL/6 background	RAW264.7, 293T	PDCD4, TNF-α, IL-10, IL-6, TGF-β, NF-κB, p38	MAPK, ERK, JNK	Upregulation of miR-16 by targeting PDCD4 could inhibit inflammatory macrophages activation in atherosclerosis through the MAPK and NF-κB pathways.	(32)
Cholesteatomas	miR-21	7 pairs of cholesteatoma and normal skin samples	–	PDCD4, IL-6R, gp130, PTEN	STAT	Upregulation of miR-21 by targeting PTEN and PDCD4 could regulate apoptosis, proliferation, invasion, and migration of Cholesteatoma.	(41)

### 2.2 Interaction Between miRNAs and PDCD in Neoplastic Disorders

#### 2.2.1 Cervical, Ovarian and Breast Cancers

A number of dysregulated miRNAs in female cancers have been shown to exert regulatory roles on PDCD genes. For instance, the oncogenic miRNAs miR-150 (44) and miR-21 (45) have been shown to inhibit expression of PDCD4, thus enhancing cell proliferation and malignant behaviors of cervical cancer cells. miR-21 has similar role in ovarian (46) and breast cancers (47). miR-421/PDCD4, miR-27a-3p/PD-L1, miR-424/PD-L1, miR-26a-5p/PDCD10 and miR-26b-5p/PDCD10 are other routes of participation of miRNAs in the pathogenesis of breast cancer (**Table 4**).

**Table 4:** Interaction between miRNAs and PDCD in female cancers.

Cancer	miRNA	Animal-human	Cell lines	Targets	Pathways	Function	Ref
Cervical Cancer (CC)	miR-150	50 pairs of CC and adjacent normal tissues	HeLa, SiHa	PDCD4	–	Upregulation of miR-150 by targeting PDCD4 could enhance cell migration, invasion, and proliferation.	(44)
CC	miR-21	–	C−33A, CaSki, SiHa, HeLa, ME−180, H8	PDCD4, Bcl-2, Bax, c-myc, PTEN	AKT	Downregulation of miR−21 by targeting PDCD4 and regulating the PTEN/AKT could inhibit cell proliferation and colony formation in CC cells.	(48)
CC	miR-21	–	HeLa	PDCD4	–	Upregulation of miR-21 by targeting PDCD4 could enhance cell proliferation in CC cell lines.	(45)
Ovarian Serous Carcinoma (OSC)	miR-21	14 OSC, 14 serous cystadenoma (CA), and 14 normal ovaries from cases of uterine prolapse	–	PDCD4	–	Upregulation of miR-21 by targeting PDCD4 could result in OSC oncogenesis.	(46)
Ovarian Cancer (OC)	miR-182	13 OC tissues and 2 normal control tissues	T29, T80, OVCAR3, SKOV3, OV2008, HEY, 3AO, A2780, HO8910, C13	PDCD4	–	Upregulation of miR-182 by targeting PDCD4 could enhance cell growth, invasion, and chemoresistance in human OC.	(49)
Breast Cancer (BCa)	miR-21	60 BCa tissues and blood samples and blood samples from 30 normal volunteers	–	PDCD4	–	Upregulation of miR-21 by translational repression of the PDCD4 could promote breast cell transformation and the development of BCa.	(47)
BCa	miR-421	52 BCa tissue samples and 52 normal tissue samples	MCF-7, MDA-MB-231, Hs578bst	PDCD4	–	Downregulation of miR−421 by targeting PDCD4 could reduce cell proliferation, migration potential, and invasiveness, and enhance apoptosis in BCa cell lines.	(50)
BCa	miR-27a-3p	26 pairs of BCa and adjacent normal tissues	MCF-7, BT474, MDA-MB-23, MCF-10A, THP-1	PD-L1, MAGI2, PTEN, GRP78, PERK, ATF6, IRE1α, IL-2	PI3K/AKT	Upregulation of miR-27a-3p by targeting PD-L1 through MAGI2/PTEN/PI3K axis could enhance immune evasion in BCa.	(51)
BCa	miR-26a-5p, miR-26b-5p	Male nude mice/Human: 20 pairs of BCa and adjacent normal tissues	T24, 5637	PDCD10	–	Upregulation of miR-26a-5p and miR-26b-5p by regulating PDCD10 could suppress the BCa cell lines proliferation.	(52)
Triple-Negative Breast Cancer (TNBC)	miR-424-5p	Female Balb/c athymic nude mice	MCF10A, BT474, HCC1500, 293T, HCC1806, HCC1954, MM231	PD-L1	–	Upregulation of miR-424-5p by targeting PD-L1 could enhance the secretion of pro-inflammatory cytokines, reduce the secretion of anti-inflammatory cytokines and boost the apoptosis of tumor cells.	(53)

#### 2.2.2 Gastrointestinal Cancers

Two independent studies in esophageal squamous cell carcinoma have shown up-regulation of the PDCD4-interacting miRNA miR-183 in this type of cancer (54, 55). Up-regulation of this miRNA has resulted in down-regulation of PDCD4 expression, thus promoting proliferation and invasion of esophageal squamous cell carcinoma cells. Suppression of the PI3K/Akt signaling by LY294002 could increase PDCD4 expression and decrease miR-183 level in these cells (54). In this type of cancer, the inhibitory role of miR-21 on PDCD4 has been verified both *in vitro* and *in vivo* (56).

In gastric cancer, miR-21 has been found to affect carcinogenesis through interacting with PDCD4 (57) as well as PD-1/PD-L1 pathway (58). miR-940, miR-208a-3p, miR-23a/b, miR-129-1-3p, miR−46146, miR-503, miR-181b and miR-148a-3p are other miRNAs that participate in the pathogenesis of gastrointestinal cancers *via* regulation of expression of PDCD genes (**Table 5**).

**Table 5:** Interaction between miRNAs and PDCD in gastrointestinal cancers.

Cancer	miRNA	Animal-human	Cell lines	Targets	Pathways	Function	Ref
Oesophageal Squamous Cell Carcinoma (ESCC)	miR-183	32 pairs of primary ESCC, 9 pairs of oesophageal low-grade intraepithelial neoplasia (LG-IEN), 21 pairs of high-grade intraepithelial neoplasia (HG-IEN), and normal controls	Eca109, TE13	PDCD4	PI3K/AKT	Upregulation of miR-183 by reducing PDCD4 enhances ESCC cell proliferation and invasion.	(54)
ESCC	miR-183	81 pairs of ESCC and adjacent non-tumor tissues	EC109, EC9706, Het-1A	PDCD4	–	Upregulation of miR-183 by targeting PDCD4 inhibits apoptosis and enhance proliferation in esophageal cancer.	(55)
ESCC	miR-21	Male BALB/c-nu mice/Human: 50 pairs of ESCC and adjacent normal tissues	Eca109	PDCD4	–	Upregulation of miR-21 by targeting PDCD4 enhances proliferation, migration of ESCC both *in vitro* and *in vivo*.	(56)
ESCC	miR-21	70 pairs of ESCC and adjacent normal tissues	EC9706	PDCD4, MMP-2, MMP-9	JNK	Upregulation of miR-21 by targeting PDCD4 could increase migration and invasion of the cell in esophageal cancer.	(59)
Pancreatic Ductal Adenocarcinoma (PDAC)	miR-21	25 pairs of PDAC and adjacent normal tissues	MIA-Pa-Ca-2, HUP-T3, PSN-1	PDCD4	–	Downregulation of miR-21 could suppress proliferation and enhance cell death in PDAC.	(60)
Pancreatic Cancer	miR-142-5p	Female C57BL/6 mice	Panc02, 293T	PD-L1, IFN-γ, TNF-α, IL-10	–	Upregulation of miR-142-5p by targeting the PD-L1/PD-1 pathway could increase anti-tumor immunity and suppress mice pancreatic cancer growth.	(61)
Hepatocellular Carcinoma (HCC)	miR-183-5p	Male BALB/C Nude Mice/Human: 50 pairs of HCC and adjacent normal tissues	L02, Huh-6, Huh-7, SNU-449, Li-7	PDCD4	–	Downregulation of miR-183-5p could inhibit proliferation, survival, migration, and invasion of HCC cells.	(62)
HCC	miR-93	64 pairs of HCC and adjacent normal tissues	293T, mmcc-7721, Huh-7	PDCD4, A-Catenin, Ɣ-Catenin, N-Cadherin	–	Upregulation of miR-93 by Targeting Pdcd4 enhances invasion and metastasis by EMT in HCC.	(61)
HCC	miR-21	16 pairs of HCC and adjacent normal tissues	L02, Hepg2, Mhcc97h, Bel7402, Huh7	Pdcd4, Ap-1, Mmp-2/9	C-Jun	Upregulation of miR-21 by targeting PDCD4 and AP-1 could enhance migration and invasion in human HCC.	(63)
Gastric Cancer (GC)	miR-499-5p	–	SGC-7901, 293T	PDCD4	STAT	Upregulation of miR-499-5p *via* STAT3 signaling pathway could increase gastric cancer cell proliferation and invasion by targeting PDCD4.	(64)
GC	miR-21	–	SGC-7901, MKN-45	PDCD4, PTEN	–	Upregulation of miR-21 by targeting PTEN and PDCD could regulate cell growth, migration, invasion, and apoptosis in gastric cancer.	(57)
GC	miR-21	16 pairs of GC and adjacent normal tissues	Th17, Treg, PBMCs	PD-1/PD-L1, RORγt, IL-17, Foxp3, TGF-β1	–	Upregulation of miR-21 could by targeting PD-1/PD-L1 Pathway regulate the percentages of Th17 and Treg cells and the expression of RORγt and Foxp3 *In Vitro*.	(58)
GC	miR-21	105 pairs of GC and adjacent normal tissues	MKN1, MKN7, MKN45, MKN74, NUGC3, NUGC4, AZ521, KATOIII	PDCD4	–	Upregulation of miR-21 by targeting PDCD4 could result in biological aggressiveness in human GC.	(65)
GC	miR-940	Female BALB/c nude mice	MGC803, AGS, Jurkat, NCI-N87, MKN74	PDL1, Cbl-b	STAT5a	Upregulation of miR-940 by targeting PDL1 and Cbl-b/STAT5a could enhance the proliferation and migration of GC cells.	(66)
GC	miR-208a-3p	Male SCID mice/Human: 16 pairs of GC and adjacent normal tissues	MKN45, HGC-27, AGS	PDCD4, Caspase-3	–	Upregulation of miR-208a-3p by targeting PDCD4 could inhibit apoptosis in GC cell lines and enhance tumor growth in xenograft mice.	(67)
GC	miR-23a/b	Male SCID mice/Human:10 pairs of GC and adjacent normal tissues	MKN-45, AGS	PDCD4	–	Upregulation of miR-23a/b by targeting PDCD4 could increase tumor growth and inhibit apoptosis in GC.	(38)
GC	miR-129-1-3p	–	BGC-823, 293T	PDCD2	–	Upregulation of miR-129-1-3p by targeting PDCD2 could enhance BGC-823 cell proliferation.	(68)
Colorectal Cancer (CRC)	miR−46146	–	HCT116, HT29	PDCD10	–	miR-46146 acts as a mediator of oxaliplatin resistance *via* targeting PDCD10.	(69)
CRC	miR-503	30 pairs of CRC and adjacent normal tissues	SW480, FHC, HT-29, 293T, HCT116, SW620	PDCD4	–	Overexpression of miR-503 by targeting PDCD4 increases CRC cell migration and invasion.	(23)
CRC	miR-181b	14 pairs of CRC and adjacent normal tissues	SW480, Caco2, HT-29	PDCD4, IL-6	STAT	Upregulation of miR-181b by targeting PDCD4 could increase CRC cell proliferation and migration to enhance tumorigenesis and suppresses apoptosis.	(70)
CRC	miR-208a-3p	40 pairs of CRC and adjacent normal tissues	HCT116, SW480, SW620, HT-29, NCM460	PDCD4	–	Upregulation of miR-208a-3p by targeting PDCD4 could enhance CRC cell proliferation and invasion.	(71)
CRC	miR-21	Chicken embryo	LS174T	PDCD4	–	Downregulation of miR-21 by targeting PDCD4 could inhibit metastatic features of CRC cells.	(72)
CRC	miR-148a-3p	–	HCT116, SW837	PD-L1, IFNγ, IL-2	–	Upregulation of miR-148a-3p by targeting PD-L1 could restore T-cell viability in the tumor microenvironment.	(73)

#### 2.2.3 Head and Neck Cancers

In head and neck squamous cell carcinoma, miR-21 has been reported to increase cell proliferation through decreasing expression of PDCD4 (74). Another study in this type of cancer has revealed that miR-375 inhibits IFN-γ-associated surface expression of PD-L1. Moreover, JAK2 has been identified as a valid target of miR-375. In fact, the suppressive effects of miR-375 on PD-L1 have been found to depend on the JAK2/STAT1 pathway. Taken together, through attenuation of PD-1/PD-L1 axis, miR-375 can enhance cellular immune response against tumor cells (75).

In oral squamous cell carcinoma cells, miR-21 could enhance tumor cell invasion through targeting PDCD4 (76). This miRNA has a similar effect in the carcinogenic process in salivary adenoid cystic carcinoma (77). In oropharyngeal cancer cells, the primary inhibition of PDCD4 is facilitated by miR-21 while continuous inhibition of its expression is facilitated by miR-499. Besides, the single miR-21 site could provoke the same degree of expression inhibition as the three miR-499 sites (32).

In tongue cancer, miR-155 has been found to target Pdcd4 transcript and inhibit its expression. Forced up-regulation of Pdcd4 or miR-155 silencing in these neoplastic cancer cells could reduce AP-1-associated transcription of the BIC promoter and reduces expression of miR-155. In fact, expression of miR-155 is regulated by a feedback circuit between Pdcd4, AP-1, and miR-155. Up-regulation of miR-155 results in progression of this type of cancer (78). **Table 6** shows the interaction between miRNAs and PDCD in head and neck cancers.

**Table 6:** Interaction between miRNAs and PDCD in head and neck cancers.

Cancer	miRNA	Animal-human	Cell lines	Targets	Pathways	Function	Ref
Head & Neck Squamous Cell Carcinoma (HNSCC)	miR-21	–	UD-SCC-1/2, UM-SCC-9/11B/47/104, PCS-200-011	PDCD4	–	Upregulation of miR-21 by targeting PDCD4 could increase proliferation in HNSCC cell lines.	(74)
HNSCC	miR-375	–	Hp-2, FaDu	PD-L1, IFN-γ, IL-2	JAK, STAT	Upregulation of miR-375 by targeting PD-L1and JAK2/STAT1 signaling could enhance the cellular immune responses to HNSCC.	(75)
Oral Squamous Cell Carcinomas (OSCCs)	miR-21	50 OSCCs and 25 normal oral tissues	UT-SCC-15, 20A, 24A, 74A, 87, HOK	PDCD4	–	Upregulation of miR-21 by targeting PDCD4 could enhance tumor cell invasion in oral squamous cell carcinoma.	(76)
Tongue Cancer	miR-155	Female BALB/c athymic nude mouse	Hep3B, SiHa, MCF-7, SCC172, MDA-MB231, H1299, HCT116, SCC131, SCC745, SCC969, AWL	NF-κB, Bax, Bcl-2, Caspase-3/9		Upregulation of miR-155 by targeting PDCD4 is involved in the progression of tongue cancer.	(78)
Salivary Adenoid Cystic Carcinoma (SACC)	miR-21	27 SACC and 20 healthy controls	SACC-LM, SACC-83	PDCD4, STAT3	–	Downregulation of miR-21 by targeting PDCD4 could reduce tumor growth and invasion in SACC.	(77)
Oropharyngeal Squamous Cell Carcinoma	miR-499, miR-21	43 patients treated for tonsillar cancer and 17 matched normal tissues	HNSCC, SCC089, SCC003, SCC099, SCC029b, 293T	PDCD4, Dicer1, Drosha, DDX5, DGCR8	–	miR-499 and miR-21 by regulating PDCD4 could participate in the pathogenesis of oropharyngeal cancers.	(32)
Laryngeal Squamous Cell Carcinoma (LSCC)	miR-503	48 pairs of LSCC and adjacent normal tissues	AMC-HN-8, Tu-177, HaCaT, 293T	PDCD4	–	Upregulation of miR−503 by targeting PDCD4 could enhance tumor growth and invasion in LSCC.	(79)
Thyroid Cancer	miR-183	38 pairs of papillary thyroid cancer and adjacent normal tissues	TPC-1, BCPAP, K1, NPA PTC, Nthy-ori 3-1, 293T	PDCD4	–	Upregulation of miR-183 by targeting PDCD4 could enhance cell proliferation, migration, invasion, and inhibit apoptosis in TPC-1 cells.	(80)

#### 2.2.4 Lung Cancer

The inhibitory role of miR-182 on PDCD4 is involved in the modulation of sensitivity of lung cancer cells to cisplatin (81). Moreover, resistance to this chemotherapeutic agent has been shown to be reversed through suppression of miR-141 (82). Similarly, miR-21 silencing could inhibit proliferation and migration of lung cancer cells *via* changing expression of PDCD4 (83). miR-182 silencing has inhibited growth and invasive properties of lung cancer cells through modulation of PDCD4 levels (55). On the other hand, miR-103 has been shown to exert its tumor suppressive roles in lung cancer through targeting PDCD10 (84). **Table 7** shows the interaction between miRNAs and PDCD in lung cancer.

**Table 7:** Interaction between miRNAs and PDCD in lung cancer.

Cancer	miRNA	Animal-human	Cell lines	Targets	Pathways	Function	Ref
Non-Small Cell Lung Carcinoma (NSCLC)	miR-182	–	A549	PDCD4	–	Upregulation of miR-182 by targeting PDCD4 could induce chemoresistance to cisplatin in NSCLC cells.	(81)
NSCLC	miR-141	–	A549, A549/DDP	PDCD4, Caspase-3	–	Downregulation of miR-141 *via* targeting PCDP could reverse cisplatin resistance in NSCLC cells.	(82)
NSCLC	miR-21	17 patients with NSCLC and 16 matched healthy volunteers	A549	PDCD4	–	Downregulation of miR-21 by targeting PDCD4 could reduce NSCLC cell proliferation and migration.	(83)
NSCLC	miR-103	Male BALB/c nude mice/Human: 32 pairs of NSCLC and adjacent lung tissues	A549, SPC-A1, NCI-H460, H1299, PC9, 293T, 16HBE	PDCD10	–	Upregulation of miR-103 by targeting PDCD10 could suppress cell proliferation and migration in the A549 cell line and NSCLC growth *in vivo*.	(84)
Lung Cancer	miR-182	–	A549, NCI-H1299, LTEP-a-2, SPC-A-1, NHBE	PDCD4	–	miR-182 silencing could suppress cell growth and invasion in human lung adenocarcinoma cells.	(55)

#### 2.2.5 Nervous System Cancers

miR-21 has been shown to affect the carcinogenesis of malignant peripheral nerve sheath tumors (85), glioblastoma (86) and neuroblastoma (87) through modulation of PDCD4. Moreover, over-expression the PDCD4-targeting miR-96 has been shown to promote resistance of glioblastoma cells to radiotherapy (88). **Table 8** shows the interaction between miRNAs and PDCD nervous system cancers.

**Table 8:** Interaction between miRNAs and PDCD nervous system cancers.

Cancer	miRNA	Animal-human	Cell lines	Targets	Pathways	Function	Ref
Malignant Peripheral Nerve Sheath Tumor (MPNST)	miR-21	12 MPNSTs, 11 neurofibroma, 5 normal nerves	HS-Sch-2, YST-1, NMS-2	PDCD4	–	Downregulation of miR-21 by targeting PDCD4 could induce cell apoptosis of MPNST cells.	(85)
Glioblastoma (GBM)	miR-96	–	U87-MG, T98G U251-MG, A172	PDCD4		Upregulation of miR-96 by targeting PDCD4 could improve radioresistance in GBM cells.	(88)
GBM	miR-21	13 pairs of GBM and normal brain tissue samples	SNB19, U251, U87, SF767	PDCD4	–	Downregulation of miR-21 could reduce proliferation, enhance apoptosis, and suppress anchorage-independent growth in glioblastoma-derived cell lines.	(86)
Neuroblastoma (NB)	miR-21	–	SK-N-SH, SH-SY5Y, BE2C, 293T	PDCD4, PTEN	–	Downregulation of miR-21 by targeting PTEN/PDCD4 could result in SK-N-SH cell apoptosis and suppress proliferation in NB.	(87)
Retinoblastoma	miR-181b	–	HXO-RB44, HUVECs	PDCD10, HIF-1α, GATA6	–	Upregulation of miR-181b which is induced by hypoxia could increase angiogenesis of retinoblastoma cells by regulating PDCD10 and GATA6.	(89)

#### 2.2.6 Other Malignancies

**Figure 3:**
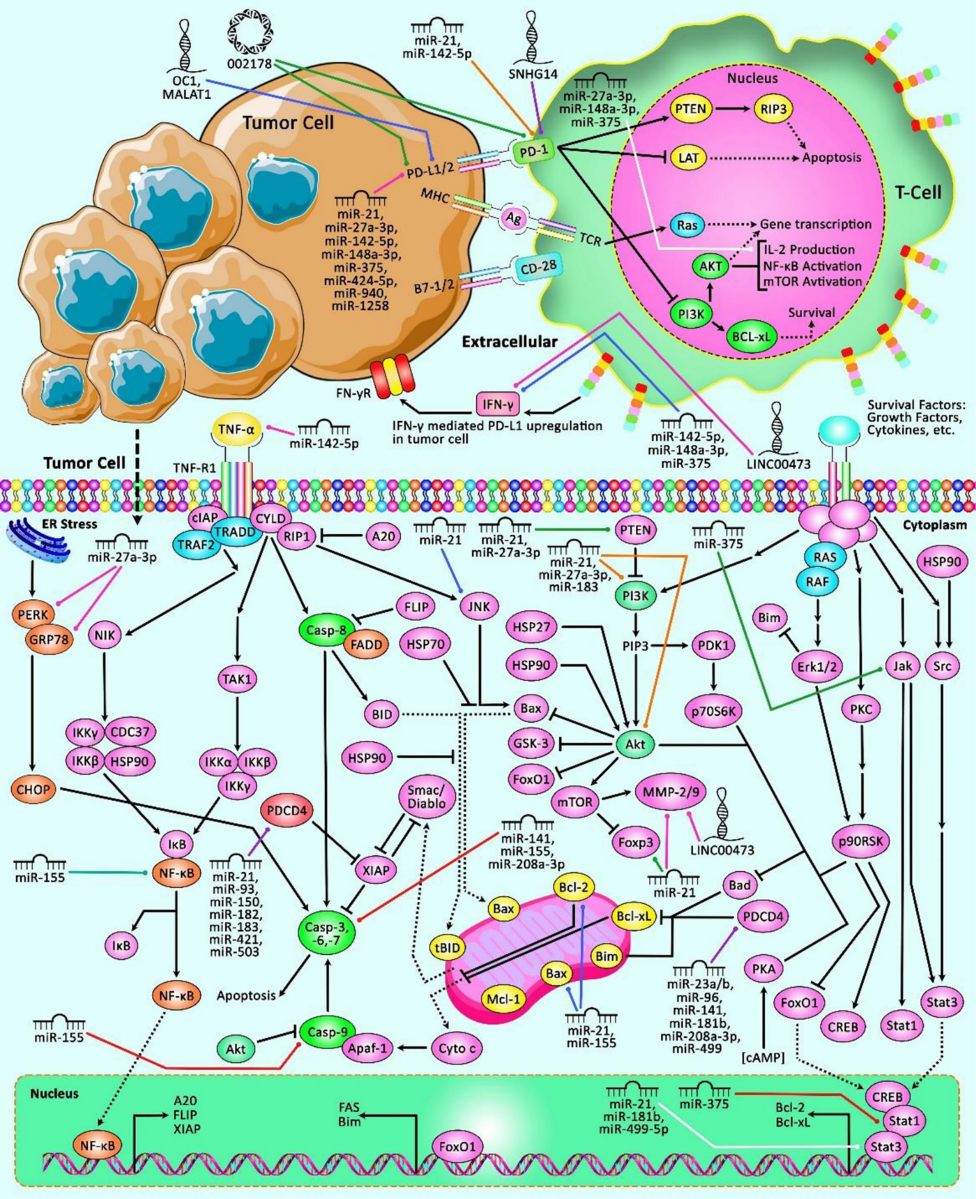
A schematic representation of the role of ncRNAs in modulating the PDCD genes in multiple human cancers. The loss of apoptotic control could cause tumor cells to survive longer and give more time for the accumulation of mutations that could, in turn, enhance invasiveness during tumor cell progression, stimulate angiogenesis, deregulate cell proliferation and interfere with differentiation (95). Apoptosis could be mediated *via* elevation or suppression of caspases. Besides, the PD-1/PD-L1 interaction could suppress T lymphocyte proliferation, survival and effector functions including cytotoxicity and cytokine release, and thereby could trigger apoptosis of tumor-specific T cells (96, 97). Previous studies have authenticated that several ncRNAs could have a crucial role in human cancers *via* interacting with various PDCD transcripts. In fact, aberrant expression of these ncRNAs could result in dysregulation of PDCDs, and therefore is associated with different kinds of cancer.

The interaction between miRNAs and PDCD has been verified in other malignancies such as hematological malignancies, bladder and kidney cancers as well as melanoma. miR-21 has been the most assessed miRNA in this regard (**Table 9**). **Figure 3** represents the role of several ncRNAs in several human cancers *via* interacting with PDCD genes.

**Table 9:** Interaction between miRNAs and PDCD in other cancers.

Cancer	miRNA	Animal-human	Cell lines	Targets	Function	Ref
Acute Myeloid Leukemia (AML)	miR-183	106 pairs of pediatric AML and normal controls	HL60, K562	PDCD6	Upregulation of miR-183 by targeting PDCD6 could enhance cell proliferation and inhibit apoptosis in pediatric AML.	(90)
Multiple Myeloma	miR-1258	20 MGUS patients, 63 with myeloma at diagnosis, and 30 myeloma patients at relapse/progression	KMS-12-PE, MOLP-8, OPM-2, U-266, NCI-H929, OCI-MY5, KMS-27, KMS-11/BTZ, OPM-2/BTZ, LP-1, RPMI-8226, JJN-3, XG-1, RPMI-8226R, WL-2, MMKKF	PDL1	Overexpression of miR-1258 could lead to reducing the expression of PD-L1 during myeloma progression.	(91)
Bladder Carcinoma (BC)	miR-21	22 patients with BC and 3 corresponding normal urothelial tissue	–	PDCD4	Upregulation of miR-21 could reduce the expression of PDCD4 in BC.	(92)
Renal Cell Carcinoma (RCC)	miR-21	–	786−O, A498, HMEC-1	PDCD4	Upregulation of miR-21 by targeting the PDCD4/c-Jun signaling pathway could increase the migration, invasion, and angiogenic abilities of RCC cells.	(93)
Malignant Melanoma	miR-21	67 pairs of human cutaneous malignant melanoma and normal nonmalignant control skin	–	PDCD4	Upregulation of miR-21 by targeting PDCD4 could enhance tumor size and metastasis in malignant melanoma.	(94)

### 2.3 Association Between PDCD-Interacting miRNAs and Cancer Prognosis

Among PDCD-related miRNAs, overexpression of miR-183-5p, miR-21 and miR-93 has been correlated with shorter overall survival of patients with different types of neoplasms. However, miR-26a-5p, miR-103 and miR-183 have been revealed to have opposite effect (**Table 10**).

**Table 10:** Association between PDCD-interacting miRNAs and cancer prognosis.

Sample	Results of Kaplan-Meier analysis	Ref
50 pairs of tumorous tissue samples and normal tissue samples	Overexpression of miR-183-5p was correlated with shorter overall survival of HCC patients.	(62)
27 cases of resected SACC and 20 healthy controls	Overexpression of miR-21 in SACC tissues was correlated with poor prognosis.	(77)
64 pairs of human primary HCC tissues and the corresponding normal tissues	Overexpression of miR-93 was correlated with shorter overall survival of HCC patients.	(61)
Male BALB/c-nu mice/Human: 50 pairs of primary ESCC and adjacent normal tissues	Overexpression of miR-21 was correlated with shorter overall survival of ESCC patients.	(56)
105 pairs of GC samples and matched controls	Overexpression of miR-21 was correlated with shorter overall survival of GC patients.	(65)
Male nude mice/Human: 20 pairs of BC tissues and corresponding adjacent bladder tissues	Low expression of miR-26a-5p was correlated with shorter overall survival of BC patients.	(52)
Male BALB/c nude mice/Human: 32 pairs of fresh primary NSCLC tissues and matched adjacent noncancerous lung tissues	Low expression of miR-103 was correlated with shorter overall survival of NSCLC patients.	(84)
106 pairs of pediatric AML and normal controls	Low expression of miR-183 was correlated with better overall survival of AML patients.	(90)

## 3 LncRNAs and PDCD

LncRNAs are transcripts with a wide range of length, i.e. 200 to thousands of nucleotides. They exert regulatory roles in different phases of gene expression through diverse action modalities (98). Limited numbers of lncRNAs have been found to affect expression of PDCDs. In fact, lncRNAs that participate in the regulation of PDCDs mostly affect expression of miRNAs and through this route they exert their function. For instance, MALAT1 has been shown to affect apoptosis of regulates cardiomyocytes through serving as a sponge for miR-200a-3p, a miRNA that inhibits PDCD4 expression. This function of MALAT1 has impacts in the cardiac injury produced by hypoxia/reperfusion in the course of myocardial infarction (98). Meanwhile, this lncRNA has been shown to affect progression of lung cancer through miR-200a-3p/PD-L1 axis (99). The known miR-21/PDCD4 axis has been shown to be controlled by two lncRNAs, namely MEG3 (90) and CiRS-126 (100), in the contexts of ischemic neuron death and PCOS, respectively.

The impact of Lnc-OC1 on PDCD-related pathways has been verified in endometrial cancer, where suppression of this lncRNA has been shown to stimulate apoptosis and decreased viability of neoplastic cells through influencing miR-34a/PD-L1 axis (69). Moreover, the lncRNA SNHG14 has been found to serve as a sponge for miR-5590-3p and enhance expression of ZEB1 to promote progression of certain type of B cell malignancy and facilitate evasion of malignant cell from immune responses through modulation of PD-1/PD-L1 checkpoint (101). Expression of this axis has also been shown to be regulated by a circular RNA, namely circRNA-002178 in lung cancer (69). Finally, LINC00473 can facilitate pathogenic course of pancreatic cancer through sponging miR-195-5p and enhancing expression of PD-L1 (102). In colorectal cancer cells, Linc00472 has been found to act as a sponge for miR-196a to release PDCD4. Besides, up-regulation of miR-196a or down-regulation of PDCD4 could reverse Linc00472-mediated suppression of cell proliferation and activation of apoptosis in these cells. Forced over-expression of Linc00472 could hinder tumor growth in animal models. Taken together, Linc00472 could suppress proliferation and induce apoptosis *via* enhancement of PDCD4 expression by decoying miR-196a (103). **Table 11** shows the interaction between lncRNAs and PDCD in different conditions.

**Table 11:** Interaction between lncRNAs and PDCD in different conditions.

Disease	LncRNA	Animal-human	Cell lines	Targets	Function	Ref
Myocardial Infarction (MI)	MALAT1	Female C57BL/6 mice	AC16	PDCD4, miR-200a-3p, IL-1, IL-8, TNF-α, Bcl-2, Bax, Cyclin-D1	Downregulation of MALAT1 by targeting the miR-200a-3p/PDCD4 axis could improve cell viability and suppress cell apoptosis in the hypoxia-induced myocardial cells.	(98)
Stroke	MEG3	Male C57BL/6 J mice	N2a	PDCD4, miR-21	Downregulation of MEG3 could protect against ischemic damages and enhance overall neurological functions *in vivo*.	(90)
Polycystic Ovarian Syndrome (PCOS)	CiRS-126	Female CF1 mice/Human: 18 PCOS patients and 5 healthy controls	–	PDCD4, miR-21, ROS, Bcl-2, Bax	Downregulation of ciRS-126 by targeting the miR-21/PDCD4 axis could decrease proliferation and enhance apoptosis in PCOS granulosa cells.	(100)
Endometrial Carcinoma (EC)	Lnc-OC1	28 pairs of EC and adjacent normal tissues	Ishikawa, HESCs	PD-L1, miR-34a	Downregulation of Lnc-OC1 by targeting PD-L1 could suppress cell growth and enhance cell apoptosis of EC cells.	(69)
Non-Small Cell Lung Carcinoma (NSCLC)	MALAT1	113 pairs of NSCLC and adjacent normal tissues	A549, CAL-12T	PD-L1, miR-200a-3p	Upregulation of MALAT1 by targeting the miR-200a-3p-PD-L1 axis could increase proliferation, mobility, migration, and invasion of NSCLC cells.	(99)
Diffuse Large B Cell Lymphoma (DLBCL)	SNHG14	BALB/c mice/Human: 38 pairs of DLBCL and adjacent normal tissues	GM12878, 293T, A20), OCI-LY7, DB, U2932, FARAGE	PD-1, ZEB1, miR-5590-3p, E-cadherin, N-cadherin	Upregulation of SNHG14 by targeting PD-1-miR-5590 axis could enhance proliferation, invasion, and EMT in DLBCL.	(101)
Lung Adenocarcinoma (LUAD)	circRNA-002178	105 pairs of LUAD and adjacent normal tissues	A549, PC9, 95D, BEAS-2B	PDL1/PD1	Upregulation of circRNA-002178 could enhance PDL1/PD1 expression in LUAD.	(69)
Pancreatic Cancer (PC)	LINC00473	134 PC patients and 20 healthy controls	SW‐1990, Panc‐1, BxPC‐3, AsPC‐1, CAPAN‐2, H6C7	PD‐L1, miR‐195‐5p, Bcl‐2, Bax, IFN‐γ, IL‐4, MMP‐2/9/10	Downregulation of LINC00473 by targeting PD‐L1 could increase apoptosis and decrease proliferation, invasion, and migration of the PC cells.	(102)

## 4 Discussion

As important regulators of apoptosis, PDCDs participate in normal development as well as several pathological conditions. Expression and function of PDCDs are under controls of several non-coding RNAs, especially miRNAs. In fact, miRNAs can affect pathoetiology of human disorders through suppression of expression of PDCDs. This type of interaction has been assessed in the context of cardiac disorders, PCOS, intervertebral disc degeneration, amyotrophic lateral sclerosis, immune thrombocytopenia, type 1 diabetes, congenital hypothyroidism, Alzheimer’s disease as well as different types of cancers. PCDC4 is the top member of this family in terms of interaction with miRNAs. In fact, miR-21, miR-208a-3p, miR-499-5p, miR-16, miR-155, miR-182, miR-141, miR-96, miR-182, miR-499, miR-503 and miR-183 have been verified as modulators of expression of PCDC4 in different tissues. Particularly, dysregulation of miR-21/PDCD4 axis has been identified as an important pathway in many diseases.

Although the roles of several PDCD-related miRNAs have been appraised in development of human cancers, data regarding their impact on patients’ survival is limited. In fact, miR-183-5p, miR-21, miR-93, miR-26a-5p, miR-103 and miR-183 are the only PDCD-related miRNAs whose association with cancer prognosis has been verified.

Since PDCDs affect an important hallmark of carcinogenesis, i.e. decreased response of cancer cells to pro-apoptotic stimuli, PDCD-related miRNAs represent an ideal group of therapeutic targets for cancers. Knock-down experiments in cell lines and animal models have revealed promising results, since down-regulation of PDCD-targeting miRNAs has substantially reduced viability of cancer cells and enhanced the response to apoptosis. These miRNAs have also been found to affect radio/chemoresistance of cancer cells, representing a novel avenue for enhancement of effectiveness of routine anti-cancer therapies.

The impact of lncRNAs on modulation of expression of PDCDs has been less studied. In fact, lncRNAs that affect expression of these proteins mainly act as molecular sponges for miRNAs. MALAT1/miR-200a-3p/PDCD4, ciRS-126/miR-21/PDCD4 and SNHG14/PD-1-miR-5590 are among lncRNA/miRNA/PDCD axes which contribute in the pathoetiology of human disorders. Thus, lncRNAs mainly affect expression of PDCD-interacting miRNAs and through this route they regulate expression of PDCDs.

## 5 Future Perspectives

Understanding the important roles of ncRNAs in regulation of function of PDCDs is expected to lead to design of specific treatments targeting these proteins *via* modulation of expression of ncRNAs. The role of PDCD5 and 7 in conjunction with ncRNAs is still unclear. These kinds of treatments can be theoretically used in a wide range of human disorders, including malignancies and disorders related with cell senescence or apoptosis. Considering the orchestrated effects of several miRNAs and lncRNAs on expressions of these proteins, targeting multiples points in this interaction network seems to be an efficient way for modulation of expressions of PDCDs.

## Author Contributions

SG-F wrote the draft and revised it. MT designed and supervised the study. HS, BH, MM, and AB collected the data and designed the figures and tables. All the authors read the draft and approved the submission version.

## Conflict of Interest

The authors declare that the research was conducted in the absence of any commercial or financial relationships that could be construed as a potential conflict of interest.

## Publisher’s Note

All claims expressed in this article are solely those of the authors and do not necessarily represent those of their affiliated organizations, or those of the publisher, the editors and the reviewers. Any product that may be evaluated in this article, or claim that may be made by its manufacturer, is not guaranteed or endorsed by the publisher.
